# Glucosamine increases hyaluronic acid production in human osteoarthritic synovium explants

**DOI:** 10.1186/1471-2474-9-120

**Published:** 2008-09-11

**Authors:** EJ Uitterlinden, JLM Koevoet, CF Verkoelen, SMA Bierma-Zeinstra, H Jahr, H Weinans, JAN Verhaar, GJVM van Osch

**Affiliations:** 1 Dept. of Orthopaedics, Erasmus MC, University Medical Centre Rotterdam, the Netherlands; 2 Dept. of Otorhinolaryngology, Erasmus MC, University Medical Centre Rotterdam, the Netherlands; 3 Dept. of Urology, Erasmus MC, University Medical Centre Rotterdam, the Netherlands; 4 Dept. of General Practice, Erasmus MC, University Medical Centre Rotterdam, the Netherlands

## Abstract

**Background:**

Glucosamine (GlcN) used by patients with osteoarthritis was demonstrated to reduce pain, but the working mechanism is still not clear. Viscosupplementation with hyaluronic acid (HA) is also described to reduce pain in osteoarthritis. The synthesis of HA requires GlcN as one of its main building blocks. We therefore hypothesized that addition of GlcN might increase HA production by synovium tissue.

**Methods:**

Human osteoarthritic synovium explants were obtained at total knee surgery and pre-cultured for 1 day. The experimental conditions consisted of a 2 days continuation of the culture with addition of N-Acetyl-glucosamine (GlcN-Ac; 5 mM), glucosamine-hydrochloride (GlcN-HCl; 0.5 and 5 mM), glucose (Gluc; 0.5 and 5 mM). Hereafter HA production was measured in culture medium supernatant using an enzyme-linked binding protein assay. Real time RT-PCR was performed for hyaluronic acid synthase (*HAS*) 1, 2 and 3 on RNA isolated from the explants.

**Results:**

0.5 mM and 5 mM GlcN-HCl significantly increased HA production compared to control (approximately 2 – 4-fold), whereas GlcN-Ac had no significant effect. Addition of 5 mM Gluc also increased HA production (approximately 2-fold), but 0.5 mM Gluc did not. Gene expression of the HA forming enzymes *HAS *1, 2 and 3 was not altered by the addition of GlcN or Gluc.

**Conclusion:**

Our data suggest that exogenous GlcN can increase HA production by synovium tissue and is more effective at lower concentrations than Gluc. This might indicate that GlcN exerts its potential analgesic properties through stimulation of synovial HA production.

## Background

Glucosamine (GlcN) is popular among patients suffering from osteoarthritis (OA). Although the efficacy of GlcN in OA is still under debate, subgroup analysis in patients with moderate-to-severe pain in a recent large NIH trial indicated that glucosamine hydrochloride (GlcN-HCl) might have a positive effect on pain [1]. Two clinical studies indicated that GlcN might have structure modifying effects on cartilage in knee OA [2,3]. The claim of structure modifying properties of GlcN is mostly based on the effects on cartilage. However, cartilage is not innervated and thus pain reduction after GlcN administration, as has been reported in the previously mentioned NIH trial, can therefore not be explained by the effect of GlcN on cartilage tissue. Considering pain reduction in OA, viscosupplementation with hyaluronic acid (HA) has been shown to have beneficial effects on both pain and function in patients with knee OA [4]. Since GlcN is an important building block of HA and HA is found in high amounts in articular joints, increasing HA production through the administration of GlcN might be a way to explain the pain relieving effect of GlcN. HA is synthesized by hyaluronic acid synthases (*HAS*) which covalently link the monomeric building blocks glucuronic acid and N-Acetyl-glucosamine (GlcN-Ac) in an alternating fashion. Hence, if the *HAS *enzymes are at hand, availability of its building blocks is another prerequisite for the synthesis of HA. But, more availability of substrate does not necessarily have to lead to more HA.

The aim our study was to evaluate whether GlcN derivatives have an effect on HA production in a human synovium explant model. Specifically, we investigated if HA production can be stimulated by adding GlcN and if GlcN affects gene expression of the *HAS *enzymes.

## Methods

### Explant culture

Human synovium was obtained from patients suffering from knee OA at time of total knee arthroplasty operation (12 patients, age 48–70 male:female ratio 1:2, with approval of the Ethical Committee number MEC 2004-140). To remove any remaining synovial fluid or blood, raw material was rinsed three times in physiological saline. From this raw material explants of approximately 3 mm^2 ^each were created with a scalpel and pooled in a Petri dish. Per condition, explants were randomly taken from the Petri dish and cultured in a 6-well plate with 3 ml culture medium per well. During the whole experiment this culture medium consisted of low glucose (1000 mg/l l = 5.55 mM) D-MEM (Gibco, Grand Island, NY), supplemented with 10% fetal calf serum (containing 0.5 mM glucose), 50 μg/ml gentamicin, 1.5 μg/ml fungizone. The total amount of explants per patient differed. Therefore, care was taken to use an equal number of explants per culture condition within one patient. In the final analysis, results were corrected for total sample weight.

After a 24 hour pre-culture period (day 1), the culture medium was refreshed and the actual experiment was started by supplementing the culture medium with different derivatives for 48 hours (day 2–3).

In the first series of experiments (N = 6 patients) the effect of different GlcN derivatives on HA production was studied. Therefore, the culture medium was supplemented with GlcN-HCl (Sigma, St. Louis, MO) or GlcN-Ac (Sigma, St. Louis, MO) at 5 mM.

In the second series of experiments (N = 6 patients) we investigated the effect of different GlcN concentrations on HA production. We also investigated the effect of glucose (Gluc) concentrations similar to that of GlcN on HA production, since *in vivo *GlcN is being formed from Gluc. Finally, in the second series of experiments the effect of GlcN and Gluc on *HAS *gene expression was studied. In this second series of experiments the culture medium was supplemented with GlcN-HCl or Gluc (Sigma, St. Louis, MO) at 0.5 mM and 5 mM, respectively. In all experiments the culture medium without GlcN or extra Gluc supplementation was used as a control.

For all experiments, at day 3 medium supernatant was stored at -20°C for analysis, explants were harvested and the wet weight per sample was determined. The explants from experiment 2 were snap frozen in liquid nitrogen for RNA isolation.

### Analysis

HA synthesis was measured in culture medium using an enzyme-linked binding protein assay (Hyaluronic Acid test Kit, Corgenix Inc., Westminster, CO) in a 96 wells plate. The wells were coated with HA binding protein from bovine cartilage to capture HA and an enzyme-conjugated version of HA binding protein to detect and measure HA in the sample using a spectrophotometer at 450 nm (650 nm reference). HA from rooster comb was used as standard. The resulting amount of HA in the culture medium was corrected for sample weight. For display purposes, these values were expressed relative to the control condition on day 3.

The wet weight per sample of the snap frozen explants from experiment 2 was determined and the frozen synovium was then processed using the Mikro-Dismembrator S^® ^(B. Braun Biotech International GmbH, Melsungen, Germany). RNA was extracted using RNA-Bee™ (TEL-TEST, Inc; Friendswood, TX, USA) according to manufacturer's guidelines and subsequently precipitated with 2-propanol. RNA was further purified using RNeasy Micro Kit (Qiagen, Venlo, The Netherlands) with on-column DNA-digestion. Total RNA was quantified accurately using a NanoDrop™ 1000 spectrophotometer (Nanodrop technologies, Wilmington, DE) according to manufacturer's instructions and 500 ng total RNA of each sample was reverse transcribed into cDNA using RevertAid™ First Strand cDNA Synthesis Kit (MBI Fermentas, Germany). TaqMan^® ^assays were performed on an ABI Prism 7000 for *HAS *1, 2 and 3 using an assay-on-demand (Applied Biosystems, Foster City, CA, order numbers *HAS1*: Hs00758053_m1, *HAS2*: Hs00193435_m1, *HAS3*: Hs00193436_m1). Tissue processing and gene expression analysis were performed using the same protocol we described earlier in our study on human osteoarthritic cartilage explants [5].

For each patient the Ct-values of each control and experimental condition were normalized to glyceraldehyde 3-phosphate dehydrogenase. Hereafter each experimental condition was expressed relative to the corresponding control condition of the same patient, according to the 2^-ΔΔCt ^method described by Livak et al. [6,7]. The resulting number of this calculation indicated whether there was an up or down-regulation of gene expression of an experimental condition compared to its paired untreated control. Thereafter, for each subset of experimental conditions the median of these numbers was calculated. For graphical display purposes only the 2^-ΔΔCt ^values were expressed as a ^10^LOG. For statistical analysis a Friedman test with post-hoc Wilcoxon signed ranks test was performed on the amount of HA in the culture medium corrected for sample weight (HA production) and on the normalized Ct values (gene expression), using SPSS 11.5.0 (SPSS Inc., Chicago, IL). A p-value ≤ 0.05 was considered to indicate statistically significant differences.

## Results

**Figure 1:**
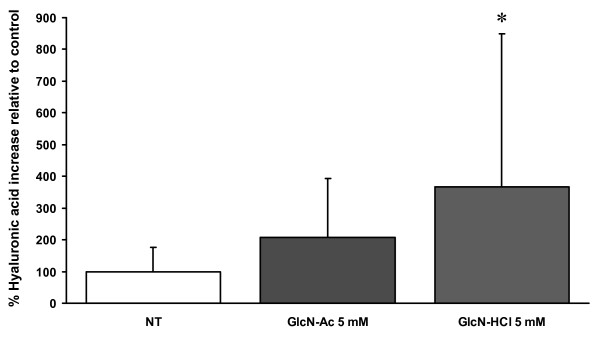
**HA after addition of 5 mM GlcN-Ac or GlcN-HCl to human synovium explants**. Amount of hyaluronic acid secreted into culture medium after 48 hours treatment of human osteoarthritic synovium explants with 5 mM GlcN-Ac (N = 6) or 5 mM GlcN-HCl (N = 6) for 2 days. Values are expressed relative to the non-treated (NT) control condition which is set at 100% (N = 6, first bar). * indicates a statistically significant difference (p = 0.028).

In the first set of experiments, the amount of HA in the medium was increased significantly 3.66 fold by the addition of 5 mM GlcN-HCl when compared to the control condition (Fig. 1). The increase after addition of 5 mM GlcN-Ac was not statistically significant.

**Figure 2:**
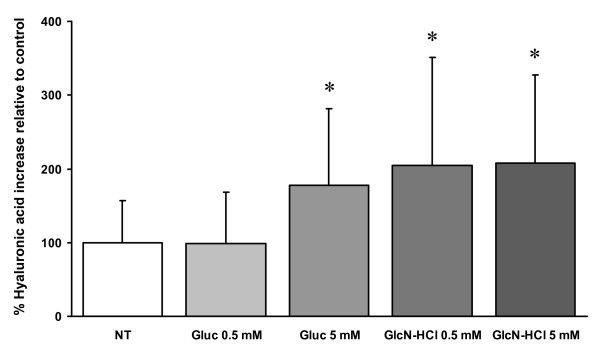
**HA after addition of 0.5 and 5 mM GlcN-HCl or Gluc to human synovium explants**. Amount of hyaluronic acid secreted into culture medium after 48 hours treatment of human osteoarthritic synovium explants with 0.5 mM GlcN-HCl (N = 6), 5 mM GlcN-HCl (N = 6), 0.5 mM Gluc (N = 5) or 5 mM Gluc (N = 6) for 2 days. Values are expressed relative to the non-treated (NT) control condition which is set at 100% (N = 6, first bar). * indicates a statistically significant difference (p = 0.028).

In the second set of experiments, in which samples from 6 other patients were used, HA in the culture medium was significantly increased by 0.5 mM GlcN-HCl (204%) and 5 mM GlcN-HCl (207%) and by addition of 5 mM Gluc (178%), but not by 0.5 mM Gluc (Fig 2).

**Figure 3:**
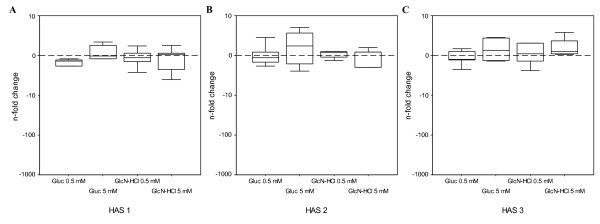
***HAS *gene expression after addition of GlcN-HCl or Gluc to human synovium explants**. Change in *HAS *gene expression in human osteoarthritic synovium explants after culture with glucosamine or glucose. Synovium explants were pre-cultured for 1 day, followed by 2 days of treatment with 0.5 mM GlcN-HCl (N = 6), 5 mM GlcN-HCl (N = 5), 0.5 mM Gluc (N = 5) and 5 mM Gluc (N = 6). The n-fold change for *HAS1 *(panel A), *HAS2 *(panel B) and *HAS3 *(panel C), normalized to glyceraldehyde 3-phosphate dehydrogenase and relative to the untreated control (indicated by the dotted line) is displayed on the vertical axis. Negative values indicate down-regulation and positive values indicate up-regulation of gene expression. No statistically significant differences were found.

Furthermore, all three *HAS *isoforms were expressed in the synovium explants. No statistically significant difference in gene expression was found for the experimental conditions, compared to the control condition (Fig. 3).

## Discussion

Addition of 0.5 mM or 5 mM GlcN-HCl to human osteoarthritic synovium explants *in vitro *led to more HA in the medium when compared to the control condition. 5 mM GlcN-Ac did not have a significant effect on medium HA concentration.

To this date several in vitro studies have been published on the possible working mechanisms of GlcN in OA. Interference with catabolic activities and stimulation of anabolic activities on chondrocytes have both been reported for GlcN in vitro. Studies investigating the influence of GlcN on enzymatic extra-cellular matrix breakdown of cartilage, with a culture system using interleukin-1, lipopolysaccharide or retinoic acid to induce catabolic effects, reported less matrix metalloproteinase activity and aggrecanase activity by addition of GlcN [8-13]. Studies considering the possible anabolic effects of GlcN proposed that addition of GlcN led to more glycosaminoglycan production by chondrocytes, since GlcN is the basic building block of glycosaminoglycan molecules [9,14-17]. These studies were performed with chondrocytes of different species.

Being a rate-limiting precursor in HA synthesis, GlcN was suggested earlier to exert its effects in OA influencing synovial HA synthesis [18,19]. However, direct scientific proof for this was still absent. To our knowledge this is the first study that investigated the effect of GlcN on human OA synovium explants.

In experiment 1 with synovium samples from 6 patients addition of 5 mM GlcN-Ac did not lead to a significant increase in medium HA. Therefore in experiment 2, in which samples from 6 other patients were used, only the effects of the derivative GlcN-HCl were tested at different concentrations and relative to Gluc treatment. In one of our earlier studies using bovine chondrocytes we also found that GlcN-Ac had a different effect on glycosaminoglycan production when compared to GlcN-HCl [20]. We speculate that this difference might be due to in the interference of the covalently bound acetyl group as opposed to the ionically bound, and therefore easy to dissolve, hydrochloride salt.

HA is found in almost all tissues, and plays key roles in many cellular processes such as migration, proliferation, differentiation and regulation of matrix organization [21]. HA is one of the main components of synovial fluid in articular joints and plays a major role in joint lubrication and in maintaining joint homeostasis [22]. In inflammatory joints, lower concentration of HA as well as lower molecular weight of HA have been described [23]. Size and concentration of HA determine the viscoelastic properties as well as regulate the inflammatory and tissue repair responses [24,25]. HA was also shown to be able to have anti-inflammatory effects on OA specific cytokines by down-regulation of tumor necrosis factor alpha, interleukin-8 [26]. HA, a polymer of alternating glucuronic acid and GlcN-Ac units is being synthesized at the inside of the cell at the plasma membrane, and then released into the extracellular matrix [27]. In accordance with Recklies et al. our results demonstrate that all 3 isoforms of *HAS*, the enzyme responsible for the synthesis of HA, are expressed by synovium [28]. Recklies et al. observed that the *HAS1 *message levels were always more abundant than that for *HAS2 *in synovial cells, and that the *HAS3 *message levels were always least abundant. In experiment 2, our mean Ct values showed a similar trend (data not shown). Addition of GlcN-HCl or Gluc did not alter *HAS *gene expression significantly. Thus, direct up-regulation of *HAS *gene expression does not seem to be the explanation for the increased amount of HA in the medium in our experiments. Since *HAS *gene expression was not affected by GlcN, apart from simply providing more building blocks, GlcN could also have played a role in HA synthesis by influencing HAS enzymatic activity or protein levels. In the current study design this was not investigated, and therefore this possibility can not be commented on.

The increased amount of HA in the culture medium might be explained by the fact that addition of GlcN-HCl simply led to more building blocks that are required for the synthesis of HA. Our results showed that a concentration of exogenous GlcN ten times lower than the medium Gluc concentration already led to significantly more HA production. This indicated a more effective and perhaps specific effect of exogenous GlcN as opposed to endogenous GlcN that is formed intracellular using medium Gluc as substrate.

The major limitation of our study was the use of relatively high concentrations of GlcN when compared to the *in vivo *situation. A recent report by Persiani et al. [29] showed that synovial GlcN concentration varied between 3 and 18 μM after oral administration at the therapeutic dose of 1500 mg once-a-day. In many earlier reports, including our own, much higher concentration have been used for in-vitro studies [5,10-13,20,30-34]. In chondrocytes it has been demonstrated that exogenous GlcN was incorporated in newly formed chondroitin sulphate when added at an equimolar concentration with Gluc in the culture medium. When Gluc concentration increased, chondrocytes preferably incorporated GlcN that was endogenously formed from Gluc [35,36]. These results suggested that in chondrocytes the GlcN to Gluc ratio played an important role in the utilization and therefore effectiveness of exogenously provided GlcN. Since we did not know whether this was also the case for synovium, we decided to use an equimolar and ten times lower GlcN concentration compared to medium Gluc concentration.

When trying to translate our in vitro results to clinical applicability of GlcN, the intra-articular GlcN concentration that can be reached after administration of GlcN to the patient is reason of concern and thus weakens the direct clinical relevance of our work. Since the first studies were performed on the effect of GlcN there has been debate on this topic. When GlcN-HCl was administered to horses in a single dose, intravenously as well as orally, in a dosage per kg bodyweight at clinically relevant levels, GlcN was still detectable in synovial fluids 6 hours after it was nearly completely cleared from the serum [37]. Although GlcN can thus be retained in joint fluid, it must still be questioned whether the levels of 0.5 mM or 5 mM GlcN will ever be reached in the joint. Therefore, future studies should look into the effect of lower GlcN concentrations on synovial HA production. Our result should be interpreted as a proof of concept on the possible working mechanism of GlcN in OA.

## Conclusion

In conclusion, the results of our experiments suggest that GlcN-HCl increases the production of HA in synovium. Our results were obtained with GlcN concentrations much higher than thus far have been reported in human synovial fluid after oral ingestion of a therapeutic dose [29]. Further studies are needed to investigate the effect of GlcN concentrations more likely to be reached in the human joint, before any decision can be made upon the clinical relevancy of GlcN ingestion on synovial HA production.

## Competing interests

The authors declare that they have no competing interests.

## Authors' contributions

EJU and GJVMvO conceived of the study, developed the design of the explant culture system and wrote the article. JLMK conducted the culture experiments, performed the analysis and contributed to the content of the article. CFV, HJ and JANV contributed to the design of the study and the content of the article. HW and SMABZ contributed to the content of the article. All authors read and approved the final manuscript.

## Pre-publication history

The pre-publication history for this paper can be accessed here:


